# Multifunctional Vertical Bulk‐Heterojunction Transistors With Amorphous GaO_x_ for Neuromorphic Perception‐to‐Decision

**DOI:** 10.1002/advs.78415

**Published:** 2026-10-20

**Authors:** Jinkui Si, Qiong Zhuang, Rengjian Yu, Junling Zhou, Lan Yang, Changjie Zhou, Huili Zhu, Minshuai Wang, Xiaomei Cai, Qiubao Lin, Wangying Xu, Shuqiong Lan, Huipeng Chen

**Affiliations:** ^1^ Department of Physics School of Science Jimei University Xiamen People's Republic of China; ^2^ Institute of Optoelectronic Display National & Local United Engineering Lab of Flat Panel Display Technology Fuzhou University Fuzhou People's Republic of China

**Keywords:** convolutional spiking neural networks, gallium oxide, multifunctional neuromorphic devices, organic field‐effect transistors, sensing‐memory‐computing integration

## Abstract

Multifunctional neuromorphic hardware has emerged as a promising paradigm to transcend the physical separation of sensing, memory, and computing inherent in traditional von Neumann architectures. The central challenge lies in developing integrated devices with high functional density for in‐situ fusion of sensing, memory, and computing. Here, we propose a multifunctional vertical bulk‐heterojunction transistor (MVBT) based on amorphous GaO_x_ dielectric layer, integrating four functions within a single device: deep‐ultraviolet (DUV) photodetection, threshold neuronal firing, broadband optoelectronic synaptic function, and reconfigurable logic gates. In the two‐terminal (gate‐source) mode, the ultraviolet‐selective photoresponse and electrochemical reactions of amorphous gallium oxide (a‐GaO_x_) enable deep‐ultraviolet photodetection and neuronal firing, respectively. In the three‐terminal mode, the organic bulk‐heterojunction active layer supports broadband optoelectronic synaptic function from ultraviolet to near‐infrared wavelengths. Asymmetric interfacial carrier‐injection barriers further enable bias‐reconfigurable AND/OR logic functions. In addition, a pixelated MVBT array demonstrates dual‐mode image sensing and visual preprocessing. Building upon these multifunctional capabilities, an MVBT‐based convolutional spiking neural network (CSNN) hybrid simulation architecture achieves a fingerprint recognition accuracy of 97.4%. This work provides a new strategy for constructing high‐functional‐density neuromorphic hardware integrating sensing, memory, computing, and decision‐making.

## Graphical abstract

A multifunctional vertical bulk‐heterojunction transistor (MVBT) with amorphous GaO_x_ integrates four functionalities within a single neuromorphic device. Gate‐source operation enables 254 nm deep‐ultraviolet sensing and threshold neuronal firing, whereas three‐terminal operation supports broadband UV–vis‐NIR synaptic functions and bias‐controlled reconfigurable logic gates. Moreover, MVBT‐based convolutional spiking neural network (CSNN) hybrid simulation architecture is demonstrated, achieving a fingerprint recognition accuracy of 97.4%.

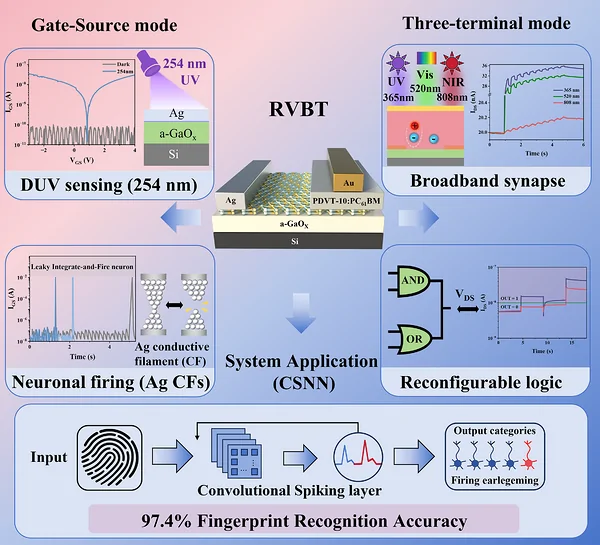

## 1 Introduction

Artificial visual systems, as a branch of artificial intelligence, have been widely applied in image recognition, autonomous vehicles, robotics, and edge artificial intelligence [1, 2, 3, 4]. Beyond merely transducing optical signals into electrical information, these systems must also rapidly respond and process to dynamic visual inputs. However, conventional von Neumann architectures characterized by physically separated processing and memory units face fundamental limitations in achieving real‐time, high‐accuracy processing of continuous, high‐throughput input signals [5, 6, 7, 8]. In contrast, the biological visual system, comprising the retina, synaptic neural networks, and the visual cortex, offers an evolutionarily optimized paradigm for overcoming this bottleneck [9, 10, 11, 12]. Specifically, optical stimuli are first captured and transduced into neural spikes by the retina. These spikes are then relayed via the optic nerve, dynamically modulated by synaptic networks, and ultimately processed in the visual cortex (Figure 1a). Drawing inspiration from this hierarchical, event‐driven, and energy‐efficient architecture, bioinspired artificial visual systems present a promising pathway toward efficient, real‐time visual signal processing [13, 14, 15, 16].

**FIGURE 1:**
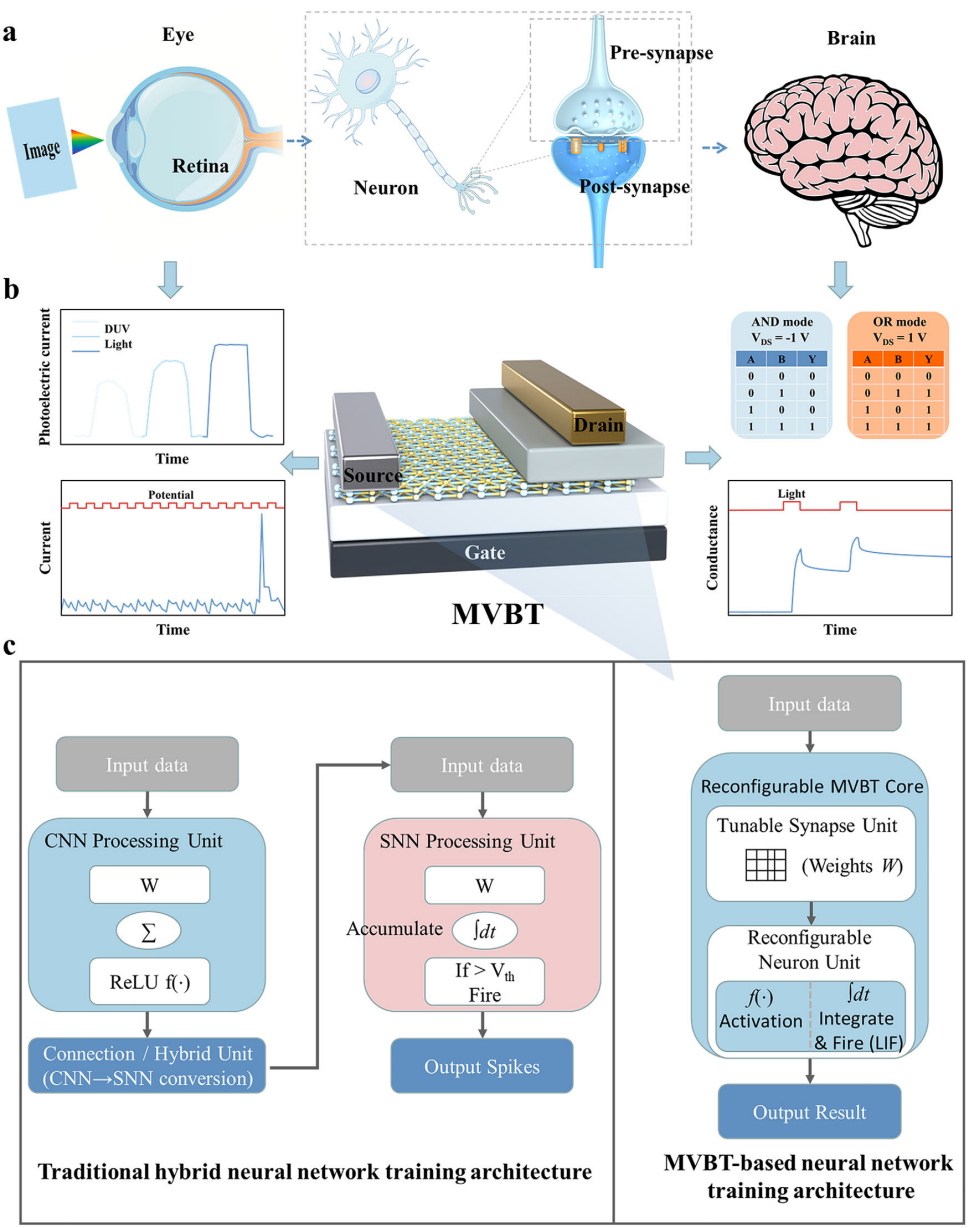
Conceptual framework of MVBT‐based neuromorphic visual system. a) Schematic illustration of the biological visual neural network. b) Four operating modes of the MVBT: photodetection, threshold firing, synaptic functionality, and reconfigurable logic gates. c) Block diagrams comparing the conventional hybrid neural network training architecture with the MVBT‐based training architecture.

Therefore, inspired by the hierarchical information‐processing pathway of biological vision, substantial research efforts have been devoted to developing artificial visual devices that perform complementary functions along the visual‐processing pathway [17, 18, 19, 20]. At the system level, combining these complementary functions often relies on the physical cascading or heterogeneous integration of multiple devices. For example, Dang et al. constructed a multi‐phototransistor–one‐memristor array for reconfigurable in‐sensor visual processing, supporting analog machine‐learning and spiking‐neural‐network tasks [21]. Although such multi‐device integration enables versatile visual information processing, the required interconnections and coordination among functional components can increase hardware complexity, latency, and energy overheads [17, 21, 22]. To reduce these integration requirements, reconfigurable devices capable of performing different functions within a single device architecture have attracted increasing attention [18, 22, 23, 24, 25, 26]. For example, Shao et al. demonstrated a Zr‐CsPbI_3_ nanocrystal‐based optoelectronic transistor with optically reconfigurable synaptic and nonvolatile memory functions [24], while Huang et al. reported a multifunctional photoelectric memristor with bias‐selectable volatile and nonvolatile operating modes [22]. However, existing single‐device reconfigurable approaches generally focus on selected functional subsets or specific operating modes, while broader integration across distinct stages of visual information processing remains limited [22, 23, 24, 25, 26]. Therefore, a compact device architecture capable of integrating complementary functions across multiple stages of visual information processing within a single physical platform is highly desirable.

Here, we developed a multifunctional vertical bulk‐heterojunction transistor (MVBT) based on amorphous gallium oxide (a‐GaO_x_) dielectric layer, which integrates four operation modes within a single physical unit: deep‐ultraviolet detection, neuronal threshold firing, broadband optoelectronic synaptic plasticity, and rectifying/logic output (Figure 1b). In the two‐terminal mode, the a‐GaO_x_ provides deep‐ultraviolet‐selective response and threshold‐switching behavior, corresponding to solar‐blind ultraviolet perception and neuron‐like firing, respectively. In the three‐terminal mode, the PDVT‐10:PC_61_BM bulk‐heterojunction layer provides broadband photoresponse and supports broadband optoelectronic synaptic plasticity. The asymmetric interfacial carrier‐injection barrier further endows the device with rectifying characteristics and reconfigurable logic‐output capability. As a result, MVBT establishes a continuous functional chain from information input and signal encoding to state modulation and output‐related operations within a single physical unit. A 4 × 5 pixelated MVBT array further demonstrates preliminary image sensing and visual preprocessing capability. Furthermore, we constructed a hardware‐inspired convolutional spiking neural network (CSNN) demonstrating the architectural advantage of MVBT for visual pattern recognition (Figure 1c), and achieved a recognition accuracy of 97.4% in a fingerprint recognition task. This work provides a novel strategy and structural paradigm for constructing highly integrated sensing‐processing neuromorphic systems.

## 2 Results

### 2.1 Device Structure and Characterization of MVBT

The MVBT device architecture is schematically depicted in Figure 2a. In this configuration, Si, MXene (Ti_3_C_2_T_x_), and Au serve as the gate, source, and drain electrodes, respectively. The chemical structures of poly[2,5‐bis(2‐decyltetradecyl)pyrrolo[3,4‐c]pyrrole‐1,4(2H,5H)‐dione‐alt‐5,5″‐di(thiophen‐2‐yl)‐2,2″‐(E)‐2‐(2‐(thiophen‐2‐yl)vinyl)thiophene] (PDVT‐10) and 6, 6]‐Phenyl‐C_61_‐butyric acid methyl ester (PC_61_BM) are presented in Figure S1. A trace amount of PC_61_BM is blended into PDVT‐10 to form bulk heterojunction, which functions as the active layer. This design eliminates the need for additional functional layers, such as floating gate, ferroelectric, or electret layers‐commonly employed to emulate synaptic behaviors. The MVBT adopts a vertical stacking configuration comprising a‐GaO_x_, MXene, and PDVT‐10:PC_61_BM. Consequently, the channel length of the active layer is defined by the thickness of the PDVT‐10:PC_61_BM active layer, whereas the thickness of the a‐GaO_x_ layer dictates the formation distance of the Ag filaments. The cross‐sectional scanning electron microscopy (SEM) image of the MVBT shown in Figure 2b reveals film thickness of approximately 50 nm for the PDVT‐10:PC_61_BM and 60 nm for the a‐GaO_x_. This confirms both the synaptic and neuronal channels of the MVBT possess ultra‐short (nanoscale) channel lengths. Figure 2c presents the absorption spectra of the various films. The MXene film exhibits strong absorption in the ultraviolet (UV) region and weak absorption in the visible region, whereas the PDVT‐10 absorbs primarily in the visible and near‐infrared (NIR) regions, and PC_61_BM absorbs across both the visible and UV regions. Owing to the complementary absorption spectra of PDVT‐10 and PC_61_BM, the blended film achieves broad absorption spanning the UV–vis‐NIR range, thereby endowing the device with broadband photoelectric response capabilities. The high capacitance of a‐GaO_x_ enables effective gate control. To evaluate the dielectric response of the a‐GaO_x_ film, the current density‐electric field characteristics (J–E) characteristics and the corresponding capacitance‐frequency (C‐f) characteristics (20 Hz‐1 MHz) of an Al/a‐GaO_x_/p^++^Si metal‐insulator‐metal (MIM) capacitor were measured [27]. As shown in Figure 2d, the results indicate that the a‐GaO_x_ dielectric layer maintains a low leakage current density in the low‐electric‐field region, while the current density increases noticeably only at high electric fields, demonstrating the good insulating property of the film. Meanwhile, the inset C‐f curve shows that the device exhibits a stable capacitance response over the measured frequency range, indicating that the a‐GaO_x_ dielectric layer possesses stable dielectric behavior and excellent insulation performance, thereby ensuring reliable and efficient gate control and conductance modulation in the device. The superior chemical and thermal stability of a‐GaO_x_ supports the reproducible growth and rupture processes of conductive filaments [28, 29, 30]. Moreover, its strong UV photoresponse of a‐GaO_x_ endows the device with high‐sensitivity photodetection capability [31, 32, 33]. Furthermore, a novel 2D conductive material MXene with excellent electrical conductivity and mechanical flexibility functions as the mesh source electrode in the MVBT. This design mitigates the randomness of conductive filament (CF) growth and enhances gate modulation [34, 35]. Figure 2e shows the X‐ray photoelectron spectroscopy (XPS) analysis of a‐GaO_x_, revealing the Ga 2p_3/2_ and Ga 2p_1/2_ doublets at 1118.2 and 1144.9 eV, respectively, as well as the O 1s peaks at 530.95 and 532.34 eV. These characteristic peaks confirm the formation of GaO_x_ film. As shown in Figure 2f, the XRD pattern of the a‐GaO_x_/Si sample is nearly identical to that of the bare Si substrate. Apart from the diffraction peak originating from the Si substrate, no obvious characteristic peaks corresponding to crystalline GaO_x_ are observed, indicating that the deposited GaO_x_ film lacks long‐range order and is therefore amorphous in nature. As shown in Figure 2g, the absorption edge of a‐GaO_x_ is located at approximately 280 nm, and the inset indicates a direct bandgap of up to 4.8 eV. Figure 2h presents the AFM image of the a‐GaO_x_ thin film. The root‐mean‐square (RMS) surface roughness is 0.212 nm, indicating a smooth surface morphology. Figure 2i presents the AFM image of the PDVT‐10:PC_61_BM blending film, which exhibits an RMS surface roughness of 0.467 nm. This smooth morphology contributes to more stable conductance modulation and consistent synaptic weight update behaviors.

**FIGURE 2:**
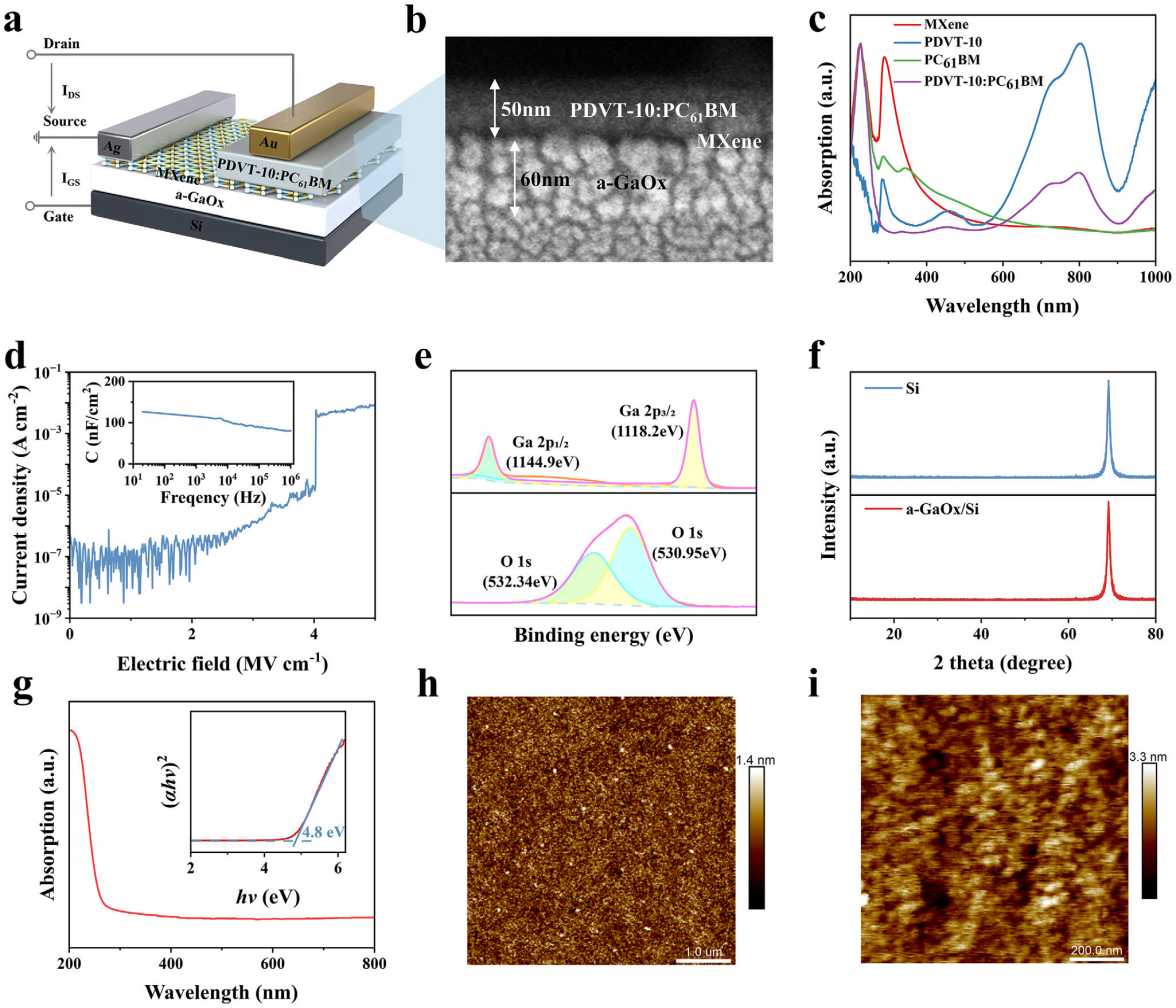
Device architecture and material characterization of the MVBT. a) Schematic illustration of the MVBT. b) Cross‐sectional SEM image of the MVBT. c) UV–vis absorption spectra of PDVT‐10, PC_61_BM, MXene, and the PDVT‐10:PC_61_BM blend films. d) Leakage current density‐electric field (J–E) characteristics of the a‐GaO_x_ dielectric layer (inset: capacitance‐frequency (C–f) characteristics). e) XPS spectra of the Ga 2p and O 1s core levels for the a‐GaO_x_ film. f) XRD patterns of the Si substrate and the a‐GaO_x_ film spin‐coated on the Si substrate. g) Absorption spectrum of the a‐GaO_x_ film. Inset: The corresponding Tauc plot. h) AFM image of the a‐GaO_x_ thin film. i) AFM image of the PDVT‐10:PC_61_BM blend film.

### 2.2 Deep‐Ultraviolet Sensing and Neuronal Dynamics in the Gate–Source Mode

Deep ultraviolet (DUV) photodetection has garnered significant research interest owing to its critical applications in flame sensing, ozone monitoring, and secure optical communication. In particular, detectors operating in the solar‐blind region (200–280 nm) benefit from negligible background interference from solar radiation at the Earth's surface, enabling ultra‐high signal‐to‐noise ratios without requiring complex optical filtering systems. To evaluate the DUV sensing capability of the MVBT, we performed an in‐depth analysis of the optoelectronic properties of the MVBT in the two‐terminal (gate–source) mode. This analysis utilized the wide bandgap property of the a‐GaO_x_ layer, which enables the selective absorption of DUV light. Devices without PC_61_BM and MXene were also fabricated and measured under identical 254 nm illumination (Figure S3). The PC_61_BM‐free device retained a pronounced DUV photoresponse comparable to that of the complete MVBT device and a clear photoresponse was also observed in the MXene‐free device. Combined with the strong DUV absorption of a‐GaO_x_ (Figure 2g), these results indicate that the 254 nm photoresponse is predominantly associated with the a‐GaO_x_ layer. Figure 3a presents the current‐voltage (I–V) characteristics of the device in the two‐terminal mode under dark conditions and 254 nm illumination (power density: 1.32 mW cm^−2^), revealing an ultra‐low dark current of less than 0.1 nA. Figure 3b shows the transient photoresponse under 254 nm light irradiation at a gate voltage (V_GS_) of 4 V. The photocurrent increases as the light intensity increases. To further elucidate the impact of gate modulation on the photodetection performance, Figure 3c displays the real‐time photocurrent response under dynamic optical switching (254 nm, 1.32 mW cm^−2^) at various V_GS_. The photocurrent is significantly enhanced with the increased positive gate voltage, aligning with the I–V characteristics observed in Figure 3a. Response speed is a critical performance metric, as it directly determines photodetector bandwidth. The rise time (τ_r_) and fall time (τ_f_) are defined as the time intervals for the photocurrent to increase from 10% to 90% of its peak value and to decrease from 90% to 10% of its peak value, respectively. As shown in Figure S2a, the device yields a τ_r_ of 36.14 ms and a τ_f_ of 17.55 ms. Figure S2b plots the photocurrent as a function of optical power. Linear fitting confirms an excellent linear relationship across different light intensities, supporting front‐end DUV sensing and feature extraction. To quantitatively assess the photodetection performance of the device, two key figures of merit were evaluated: responsivity (R) and specific detectivity (D*) [32, 36]. The dependence of both R and D* on light intensity is illustrated in Figure S2c. Responsivity is defined as the photocurrent generated per unit of incident optical power over the effective area, calculated as:

(1) $$R=\frac{I_{ph}}{P\times S}$$

where I_ph_ denotes the photocurrent induced by incident light, P represents the incident optical power density, and S is the effective illuminated area. Owing to the linearity relationship between photocurrent and optical power, responsivity remains nearly constant across the measured range, with a maximum value of 0.278 A W^−1^. The result indicates that the MVBT maintains stable photoelectric conversion capability under different illumination intensities. In addition to responsivity, detectivity, a parameter characterizing the ability to detect weak optical signals‐was evaluated using the following equation:

(2) $$D^{*}=\frac{RA^{1/2}}{{\left(2qI_{\mathrm{dark}}\right)}^{1/2}}$$

where A is the effective area of the device, q is the elementary charge, and I_dark_ is the dark current. The device achieves a peak detectivity of 5.8 × 10^9^ Jones, indicating the capability of the device to detect weak DUV illumination. Table S1 compares the results presented in this work with those of GaO_x_‐based ultraviolet photodetectors reported in recent years. These results collectively demonstrate that the MVBT exhibits highly sensitive deep‐ultraviolet photodetection in the two‐terminal mode, providing a solid foundation for its subsequent neuromorphic and multifunctional operations.

**FIGURE 3:**
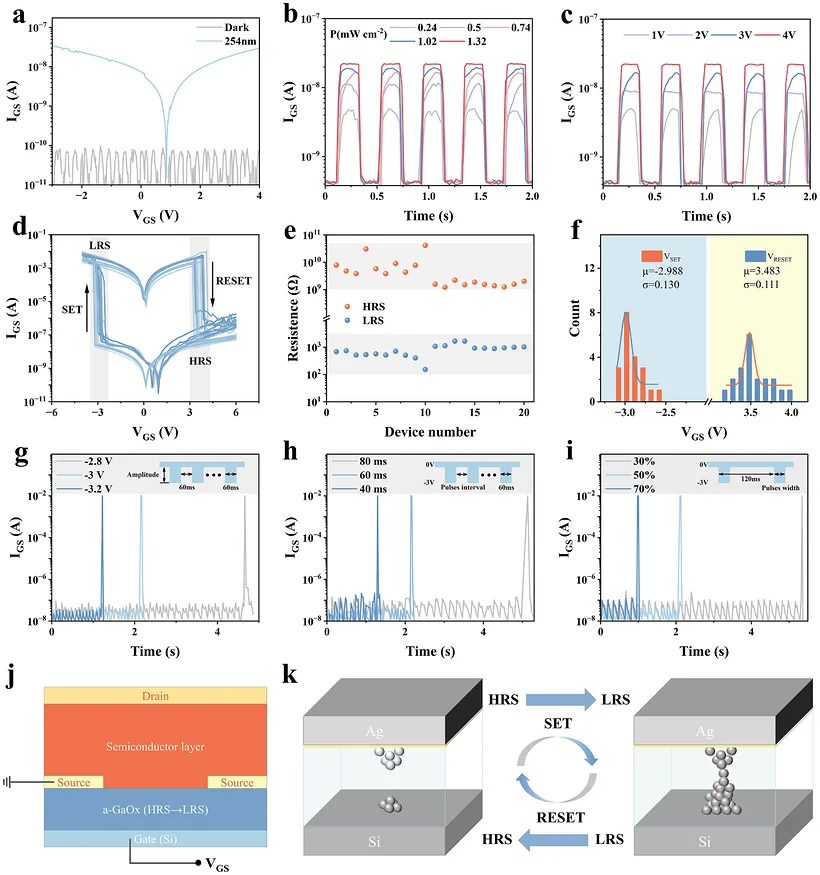
Deep‐ultraviolet sensing and neuronal dynamics of the MVBT in the gate–source mode. a) I–V characteristics of the device measured both under dark and illuminated conditions. b) Real‐time photocurrent response of the device under dynamic optical switching with varying power densities of 254 nm light at V_GS_ = 4 V. c) Real‐time photocurrent response under dynamic optical switching at various V_GS_ under 254 nm illumination with a power density of 1.32 mW cm^−2^. d) Threshold switching characteristics of the MVBT. e) Resistance distributions measured from 20 distinct MVBT devices. f) Statistical distribution of V_SET_ and V_RESET_ across 20 distinct MVBTs. g–i) Integrate‐and‐fire behavior of the MVBT. The firing latency of the neuristor channel is modulated by: g) pulse amplitude (−3.2, −3, and −2.8 V), h) pulse interval (40, 60, and 80 ms), and i) duty ratio (70%, 50%, and 30%). j) Schematic illustration of the Leaky Integrate‐and‐Fire (LIF) neuron functionality within the MVBT. k) Physical mechanism underlying the resistive switching process in the MVBT device.

Figure 3d shows the stable bidirectional threshold switching characteristics of the device's neuristor channel in two terminal (gate–source) mode. Upon V_GS_ reaching the set voltage (V_SET_), the a‐GaO_x_ layer undergoes a transition from a High Resistance State (HRS) to a Low Resistance State (LRS), accompanied by a dramatic increase in gate–source current (I_GS_) from approximately 10^−8^ A to 10^−2^ A. Conversely, when V_GS_ exceeds the reset voltage (V_RESET_), the a‐GaO_x_ layer reverts from the LRS to the HRS. Figure 3e presents the resistance distribution of the HRS and LRS across 20 distinct neuristor channels. As shown in Figure 3f, the statistical distributions of V_SET_ and V_RESET_ for these 20 channels are observed, confirming robust uniformity and stability of the device. In addition, 50 consecutive SET/RESET switching cycles were measured for a representative MVBT (Figure S4). Stable bidirectional threshold switching was maintained throughout the cycling test without obvious systematic drift in V_SET_ or V_RESET_. Together with the device‐to‐device distributions in Figure 3e,f, these results demonstrate good switching stability and reproducibility. We also used another commonly used mesh‐like electrode composed of Ag nanowires to replace MXene for comparison (Figure S5). The Ag‐nanowire‐based device retained the threshold‐switching characteristics, whereas the RESET process exhibited a more dispersed and irregular distribution. In comparison, the incorporation of MXene reduces the stochasticity associated with Ag conductive‐filament evolution and improves the controllability and reproducibility of the switching characteristics, consistent with previous reports on MXene‐containing memristive structures [35]. A more comprehensive quantitative comparison between MXene and other alternative source electrodes is provided in Table S2.

Figures 3g–i presents the electrical characterization of the device's neuronal functionalities under various programming modulation schemes. In the pulse amplitude coding mode, illustrated in Figure 3g, V_GS_ spikes of varying magnitudes (−2.8, −3, and −3.2 V) significantly influence the firing latency of the neuristor channel. Continuous stimulation with a −3.2 V spike induces channel discharge after 1.1 s. Reducing the amplitude to −3 V prolongs the integration time to approximately 2.1 s, while a −2.8 V pulse further extends the firing latency to about 4.5 s. These results highlight the precise programmability of the neuron channel through spike amplitude modulation. Notably, applying a pulse amplitude of −2.5 V elicited no discharge behavior, confirming the channel's amplitude‐dependent selectivity (Figure S6). The spike interval coding mode is demonstrated in Figure 3h. With a spike interval of 40 ms, the neuristor channel accumulates sufficient charge to fire after 1.2 s. Increasing the interval to 60 ms delays the firing event to approximately 2.1 s, and further increase to 80 ms extends the firing latency of about 4.9 s. Figure 3i demonstrates the Duty Ratio coding mode. The DR is defined by the following equation:

(3) $$DR=\frac{T_{width}}{T_{period}}\;\times 100\%$$

where T_width_ and T_period_ represent the pulse width and the signal period, respectively. At a DR of 70%, the neuristor channel fires almost immediately after an accumulation period of approximately 1 s. When the DR is reduced to 50%, the spiking trigger time delays to approximately 2.1 s, and further decreasing the DR to 30% extends the firing latency to 5.3 s. Figure 3j provides a schematic illustration of the MVBT resistive layer during operation. The fundamental mechanism underlying the spiking neuron functionality is the migration and deposition of electrochemically active metal ions within the resistive layer. Upon application of a negative voltage to the bottom silicon electrode, the top electrochemically active silver electrode undergoes oxidation, generating Ag^+^. Driven by the electric field, these ions migrate toward the Si electrode, where they are reduced to Ag atoms and subsequently accumulate (Figure 3k, left). As V_GS_ reaches V_SET_, the Ag atoms aggregate vertically to form Ag conductive filament (CF), thereby bridging the gap between the Si and Ag electrodes. This results in a sharp increase in the I_GS_ and triggers discharge of the neuron channel (Figure 3k, right). Under continuous V_GS_ pulse stimulation, the Ag CFs experience Joule heating and subsequent rupture, resulting in the leakage behavior characteristic of the neuron channel (Figure S7). The simulated potential distribution between gate and source electrode, presented in Figure S8, confirms the migration of Ag^+^ toward the gate electrode under negative V_GS_ pulses. The stochastic diffusion dynamics of Ag within the MVBT neuron channel can be described by the Langevin equation [37, 38, 39], with detailed descriptions provided in Text S1.

### 2.3 Optoelectronic Synaptic Plasticity and Working Mechanisms

Synapses play a fundamental role in governing information transmission between neurons. Under the three‐terminal configuration and appropriate electrical or optical stimulation, the MVBT operates as a synaptic device. Figure 4a and Figure S9 illustrate the transfer characteristics of the device under various source‐drain voltage (V_DS_), revealing typical p‐type transistor behavior. The output characteristics, obtained by sweeping the V_GS_ from 0 to −3 V, are presented in Figure 4b. Distinct rectifying behavior is observed. The device exhibits a rectification ratio of approximately 5 × 10^2^ at V_GS_ = 0 V. As V_GS_ increases, the rectification gradually diminishes, reaching a ratio of 2.7 at V_GS_ = −3 V. The rectification mechanism will be discussed in detail in Section 2.4. The energy band diagram shown in Figure 4c suggests that the hole injection barrier at the Au electrode/channel layer interface is lower than that at MXene/channel layer interface [34, 40, 41, 42]. Consequently, under a positive V_DS_, holes are injected from the top Au electrode, transported through the active layer, and collected by the bottom MXene layer. Figures 4d–f demonstrate the synaptic memory retention capabilities of the device. Upon applying presynaptic stimulation pulses with varying amplitudes (V_GS_ = 1, 2, 3, 4 V), both the peak and the remnant values of the drain‐source current (I_DS_) increase with the pulse amplitude (Figure 4d), successfully mimicking synaptic potentiation behavior. Conversely, applying negative presynaptic pulses (V_GS_ = −1, −2, −3, −4 V) causes both the peak and decay currents of I_DS_ to decrease as the amplitude magnitude increases, mimicking synaptic depression behavior.

**FIGURE 4:**
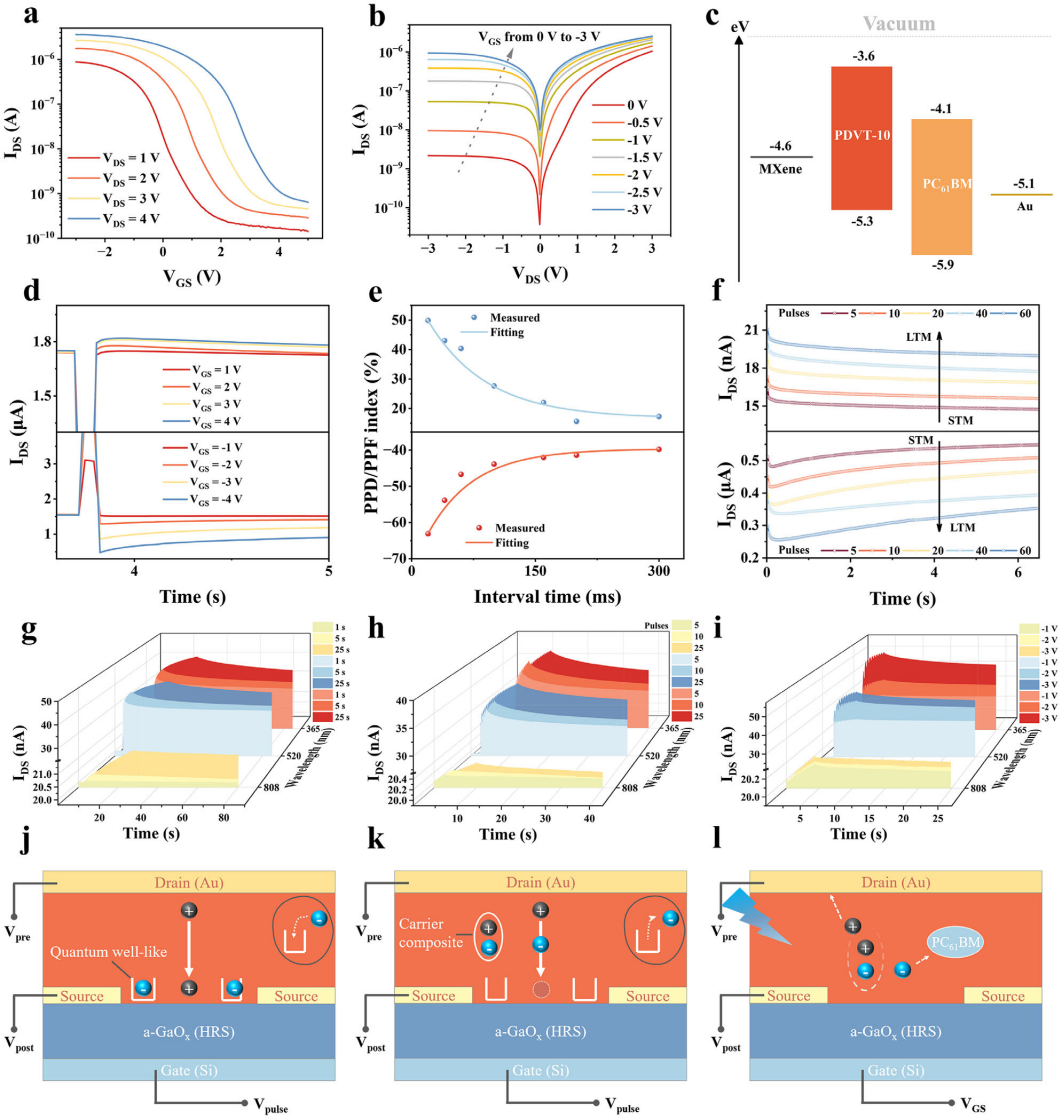
Optoelectronic synaptic plasticity of the MVBT in the three‐terminal mode. a) Transfer characteristics of the MVBT under different V_DS_. b) Output characteristics of the MVBT under various gate voltages. c) Energy level diagram of MXene, PDVT‐10, PC_61_BM, and Au. d) EPSC and IPSC responses triggered by presynaptic pulses of varying voltage amplitudes (V_GS_ = ±1, ±2, ±3, ±4 V; duration: 60 ms). e) Fitted PPF and PPD curves. f) PSC responses stimulated by pulse trains containing varying numbers of positive and negative pulses (V_GS_ = ±2 V). g–i) EPSC behaviors of the MVBT triggered by optical pulses at different wavelengths (365 nm, 520 nm, 808 nm) under an optical power density of 0.18 mW cm^−2^. g) EPSC as a function of optical pulse duration. h) EPSC as a function of the number of optical pulses. i) Voltage‐dependent EPSC behaviors triggered by 10 consecutive optical pulses (duration: 0.2 s, interval: 0.2 s). j–l) Schematic illustrations of the synaptic mechanisms within the MVBT under j) potentiation, k) depression, and l) illumination.

Paired‐Pulse Facilitation (PPF) is a classic form of short‐term plasticity in neurobiology. When two consecutive excitatory stimuli are applied, the Postsynaptic Current (PSC) triggered by the second pulse (C_2_) is larger than that evoked by the first pulse (C_1_), a phenomenon that dependent on the pulse interval (Δt). In contrast, when the second response is smaller than the first, the phenomenon is referred to as Paired‐Pulse Depression (PPD). The PPF and PPD responses of the MVBT are presented in Figure S10, with the corresponding index defined as:

(4) $$PPF/PPD\;index=\frac{C_{2}-C_{1}}{C_{1}}\;\times 100\%$$

As presented in Figure 4e, the dependence of the PPF/PPD index on the pulse interval can be fitted by a double‐exponential decay function:

(5) $$PPF/PPD\;index=A_{1}\;\exp\left(-\frac{\Delta t}{\tau_{1}}\right)+A_{2}\exp\left(-\frac{\Delta t}{\tau_{2}}\right)+A_{3}$$

where A_1_ and A_2_ are constants, τ_1_ and τ_2_ are characteristic relaxation times, and A_3_ represents the asymptotic index as Δt approaches infinity. PPF and PPD curves at various intervals are provided in Figure S11. Subjecting the biological presynaptic terminal to continuous pulse stimulation enhances synaptic weight, a key process underlying the transition from Short‐Term Memory (STM) to Long‐Term Memory (LTM). Figure 4f illustrates the evoked excitatory postsynaptic currents (EPSC) and inhibitory postsynaptic currents (IPSC) in response to pulse trains comprising 5, 10, 20, 40, and 60 electrical pulses (positive pulses: 2 V 60 ms; negative pulses: −2 V 60 ms). The EPSC magnitude progressively increases (and IPSC decreases) as the pulse number increases. The complete current‐time responses are shown in Figure S15. This pattern is consistent with the transition from STM to LTM. We further evaluated PPF under optical stimulation.

The relationship between the optical pulse interval (Δt) and the PPF index at wavelengths of 365 nm, 520 nm, and 808 nm is presented in Figure S12. Figure 4g displays the PSC triggered by single optical pulses of varying wavelengths and durations, revealing that EPSC amplitude increases with pulse duration across all wavelengths. Similarly, Figure 4h depicts the dependence of EPSC on the pulse number (5, 10, 25), confirming cumulative potentiation. To explore the influence of V_DS_ on the synaptic plasticity, we measured the change in EPSC (ΔEPSC) evoked by 10 consecutive light pulses (200 ms) across a voltage range from −1 to −3 V (Figure 4i). The results indicate that V_DS_ can be utilized to modulate memory retention characteristics. The energy consumption per synaptic event (E) can be calculated as:

(6) $$E=V_{DS}\;\times I_{DS}\times \Delta t$$

where I_DS_ denotes the peak EPSC and t is the pulse duration. Figure S13 shows the EPSC response of the MVBT device under 365 nm illumination with an intensity of 0.18 mW cm^−2^. When V_DS_ is fixed at 1 µV and t is set to 20 ms, the PSC current reaches 7.85 × 10^−10^ A, corresponding to an energy consumption of 15.7 aJ per synaptic event. Table S3 shows the comparison between MVBT and other photoelectric synaptic devices. Furthermore, by applying a train of 40 optical pulses (pulse duration: 0.2 s), we successfully simulated human learning behavior, as shown in Figure S14. The corresponding training conductance evolution under 50 excitatory pulses and 50 inhibitory pulses is presented in Figure S16. Under dark conditions, the synaptic operation of the MVBT is governed by two complementary mechanisms. First, the gate electric field induces carrier accumulation at the mesh‐like source apertures, thereby modulating the conductive channel through the semiconductor under V_DS_. Second, the incorporation of a small amount of n‐type PC_61_BM into the p‐type PDVT‐10 matrix forms local PDVT‐10/PC_61_BM/PDVT‐10 quantum‐well‐like configurations, in which the lower‐lying LUMO of PC_61_BM provides energetically favorable electron‐trapping sites [39, 43, 44]. Under positive V_GS_, the gate‐induced band bending favors electron accumulation and the filling of the localized PC_61_BM‐associated trapping states. The captured electrons reduce the mobile electron population and favor hole‐dominated transport in the synaptic channel, thereby producing an EPSC (Figure S17a). Conversely, under negative V_GS_, the direction of the gate‐induced band bending is reversed, suppressing electron accumulation and favoring the emptying of the occupied trapping states. The released electrons can subsequently recombine with holes in the PDVT‐10‐rich phase, suppressing the hole current and producing an IPSC (Figure S17b). Using COMSOL Multiphysics simulations, the current density distribution under positive and negative gate voltages were obtained, as shown in Figure S18. Figure 4l illustrates the synaptic mechanism under illumination. Upon light illumination, the PDVT‐10:PC_61_BM bulk heterojunction generates photogenerated electron‐hole pairs. Owing to the built‐in electric field at the donor‐acceptor interfaces, these charge carriers are efficiently separated, with electrons being trapped by the acceptor (PC_61_BM) domains and holes remaining in the PDVT‐10‐rich transport pathway. The trapped electrons induce a relative increase in hole concentration and enhance the channel conductance, thereby generating an EPSC, as illustrated in Figure S17c.

### 2.4 Optoelectronic Rectification Mechanism and Reconfigurable Logic Operations

Distinct from the V_GS_‐controlled charge trapping and detrapping involved in synaptic modulation, the rectifying behavior of the MVBT is mainly governed by the asymmetric carrier‐injection conditions at the Au/active‐layer and MXene/active‐layer interfaces. Figure 5a–f illustrate the corresponding operating mechanism. Figure 5a schematically depicts hole transport under positive V_DS_. Under this condition, the energy bands of PDVT‐10 bend, thereby lowering the hole‐injection barrier (Figure 5b). Consequently, holes are efficiently injected from the Au electrode into the continuous hole‐transport pathway with PDVT‐10. These holes can propagate smoothly throughout the active layer, giving rise to a substantial hole current. Meanwhile, although a small number of electrons may be injected from MXene into PC_61_BM domains, they tend to be trapped and thus contribute negligibly to the total current. Therefore, the current is dominated by holes injected from the Au electrode. Figure 5c shows the potential distribution when the channel is turned on under positive bias. Figure 5d schematically illustrates electron transport under negative V_DS_, wherein energy band bending in PC_61_BM reduces the electron‐injection barrier (Figure 5e). In this regime, hole injection would need to occur from the MXene electrode. However, the MXene/PDVT‐10 interface exhibits an exceptionally large hole‐injection barrier, effectively suppressing hole injection. Although the Au electrode could, in principle, serve as an electron‐injecting contact, electron injection and transport remain highly inefficient. This is attributed to two key factors: (i) the Au/PC_61_BM interface intrinsically has a large electron‐injection barrier, and (ii) the PC_61_BM phase is discontinuous, rendering electron injection and percolative transport extremely difficult. Consequently, the majority carrier (holes) injection pathway is blocked, while the minority carrier (electrons) fail to establish a continuous conduction path. This combination results in a “circuit‐off” state characterized by a minimal current. Figure 5f presents the potential distribution when the channel is turned on under negative bias. The gradual reduction in the rectification ratio with increasing magnitude of the negative gate bias can be attributed to the transition from contact‐barrier‐limited transport to gate‐assisted channel transport. At V_GS_ = 0 V, the vertical current is primarily governed by the asymmetric interfacial injection barriers. When a negative gate bias is applied, the vertical electric field penetrates into the organic semiconductor through the apertures of the MXene mesh electrode and induces hole accumulation near the source side. This electrostatic modulation bends the energy levels at the MXene/PDVT‐10 interface and effectively narrows the reverse injection barrier, thereby enabling field‐assisted thermionic emission and tunneling injection. Consequently, the reverse current increases much more rapidly than the forward current, because the forward branch is already dominated by efficient Au‐assisted hole injection and becomes mainly limited by the vertical channel resistance. Therefore, the rectification ratio decreases from approximately 5 × 10^2^ at V_GS_ = 0 V to 2.7 at V_GS_ = −3 V. This result indicates that the intrinsic contact asymmetry remains present, but its rectifying effect is progressively screened or bypassed by gate‐induced carrier accumulation and field‐assisted reverse injection.

**FIGURE 5:**
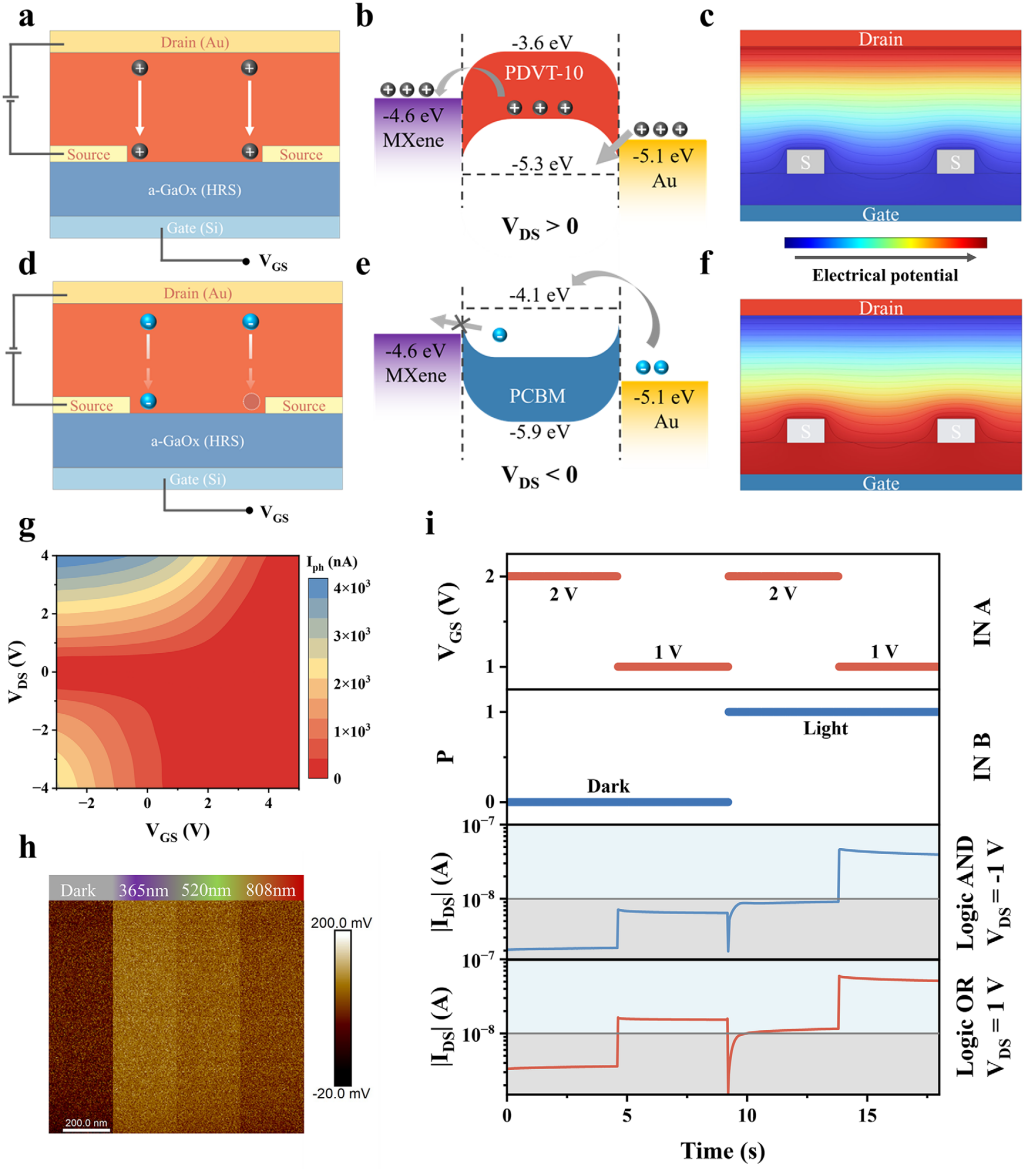
Rectifying transport mechanism and reconfigurable optoelectronic logic operation. a) Schematic illustration of hole transport under positive bias. b) Energy‐band diagram of the channel under positive bias. c) Potential distribution in the MVBT under positive V_DS_ (V_GS_ = 0 V, V_DS_ = 3 V). d) Schematic illustration of electron transport under negative bias. e) Energy‐band diagram of the channel under negative bias. f) Potential distribution in the MVBT under negative V_DS_ (V_GS_ = 0 V, V_DS_ = −3 V). g) A comprehensive 2D map of the photocurrent measured at 808 nm under various combinations of V_DS_ and V_GS_. h) Surface potential distributions of the PDVT‐10:PC_61_BM blend film under illumination spanning from the ultraviolet to the near‐infrared regions. i) A MVBT‐based switchable logic gate.

Figure 5g and Figure S19 presents a comprehensive 2D map of the photocurrent (I_ph_ = I_light_ − I_dark_) as a function of V_DS_ and V_GS_ under different illumination conditions. The map reveals a pronounced bias‐polarity‐dependent asymmetric photoresponse. This strong polarity‐dependent asymmetry is attributed to the formation of asymmetric Schottky contacts at the interfaces between the active layer and the Au electrodes, and between active layer and MXene electrodes. Such asymmetric contacts spontaneously establish an internal built‐in electric field within the active layer, directed from the Au electrode toward the MXene electrode. Physically, this built‐in field drives photogenerated holes toward the Au electrode and photogenerated electrons toward the MXene electrode, thereby constituting the fundamental basis for the device to generate a photovoltaic response. When a reverse gate bias is applied, the externally induced electric field is aligned with the built‐in field, enhancing the net internal field and thus increasing the photocurrent. In contrast, under a forward gate bias, the external field opposes the built‐in field, partially cancelling and weakening the net internal field, which results in an extremely weak photocurrent. To further verify the photoinduced interfacial potential redistribution associated with the asymmetric contacts, in situ Kelvin probe force microscopy (KPFM) measurements were performed (Figure 5h). Upon light illumination with 365 nm, the surface potential of the active layer increased to approximately 180 mV. Under 520 nm illumination, the surface potential increased to about 173 mV, whereas under near‐infrared (808 nm) illumination, the surface potential was only around 120 mV. These wavelength‐dependent surface‐potential shifts indicate light‐induced charge redistribution in the active layer and provide supporting evidence for the built‐in‐field‐assisted carrier separation, which is consistent with the bias‐/polarity‐dependent asymmetric photoresponse observed in Figure 5g and Figure S19.

Conventional logic‐gate architectures typically require at least two transistors connected in series or parallel to execute basic logic operations, which inherently constrains the achievable functional density in integrated circuits [45]. In contrast, as shown in Figure 5i, by leveraging the pronounced bias‐ and polarization‐dependent asymmetry of photoresponse in the MVBT, we realize two distinct logic functions (AND and OR) within a single device. In this configuration, the V_GS_ (denoted as Input A) and incident light (denoted as Input B) serve as dual input signals, while the output is defined by the I_DS_ (OUT). The input conditions are defined as follows: V_GS_ = 2 V represents logic “0” and V_GS_ = 1 V represents logic “1”. Without light illumination represents logic “0” and light illumination represents logic “1”. The output logic level is determined using a current threshold of 10^−8^ A, where I_DS_ > 10^−8^ A corresponds to OUT = 1 and I_DS_ < 10^−8^ A corresponds to OUT = 0. When operated at a drain bias of V_DS_ = −1 V, the MVBT exhibits AND‐gate behavior. Notably, under the same input conditions, reversing the drain bias to V_DS_ = +1 V switches the logic operation to an OR gate. This bias‐tunable switching between AND and OR logic operations demonstrate the intrinsic multifunctionality of the MVBT.

### 2.5 Dual‐Mode Visual Preprocessing in a Pixelated MVBT Array

A 4 × 5 pixelated MVBT array was fabricated for optical pattern processing. A “T”‐shaped optical pattern was projected onto the MVBT array through a patterned mask, and the output current of each pixel was read to reconstruct the spatial image (Figure 6a). Figure 6b shows the transfer characteristics of 20 MVBT pixels measured under dark conditions at V_DS_ = 3 V, exhibiting similar modulation behavior, which confirms the good functional reproducibility of the 4 × 5 array. The corresponding device‐to‐device variations in threshold voltage and ON/OFF current ratio are summarized in Figure S21. This pixel‐to‐pixel consistency provides the basis for subsequent optical image mapping and visual preprocessing demonstration. Subsequently, the MVBT array was operated in the gate–source two‐terminal mode. Under 254 nm illumination, the photocurrent of the illuminated pixels increased significantly, allowing the “T” pattern to be reconstructed from the pixel‐resolved current map. After removing the deep‐ultraviolet light, the output current rapidly decayed toward the baseline, and the encoded pattern disappeared accordingly (Figure 6c). This detector‐like transient response enables deep‐ultraviolet image extraction while suppressing residual signals, which is beneficial for distinguishing informative signals from background interference. In contrast, when the array was operated in the three‐terminal synaptic mode, 520 nm green‐light pulses were used to write the “T” pattern. With increasing pulse number, the output current of the stimulated pixels gradually increased, indicating optoelectronic synaptic potentiation. After the light was turned off, the encoded pattern could still be retained for a certain period and then gradually decayed (Figure 6d). Such a synaptic‐like persistent response enables pulse‐induced image enhancement and temporary retention, which are important functions of synaptic visual preprocessing. Therefore, the 4 × 5 pixelated MVBT array demonstrates dual‐mode image sensing and preprocessing capability within the same device platform. The two‐terminal mode enables transient deep‐ultraviolet image extraction and background refreshing, whereas the three‐terminal synaptic mode enables visible‐light‐induced image enhancement and temporary memory. This shows the potential of the MVBT array for optical sensing and neuromorphic computing applications.

**FIGURE 6:**
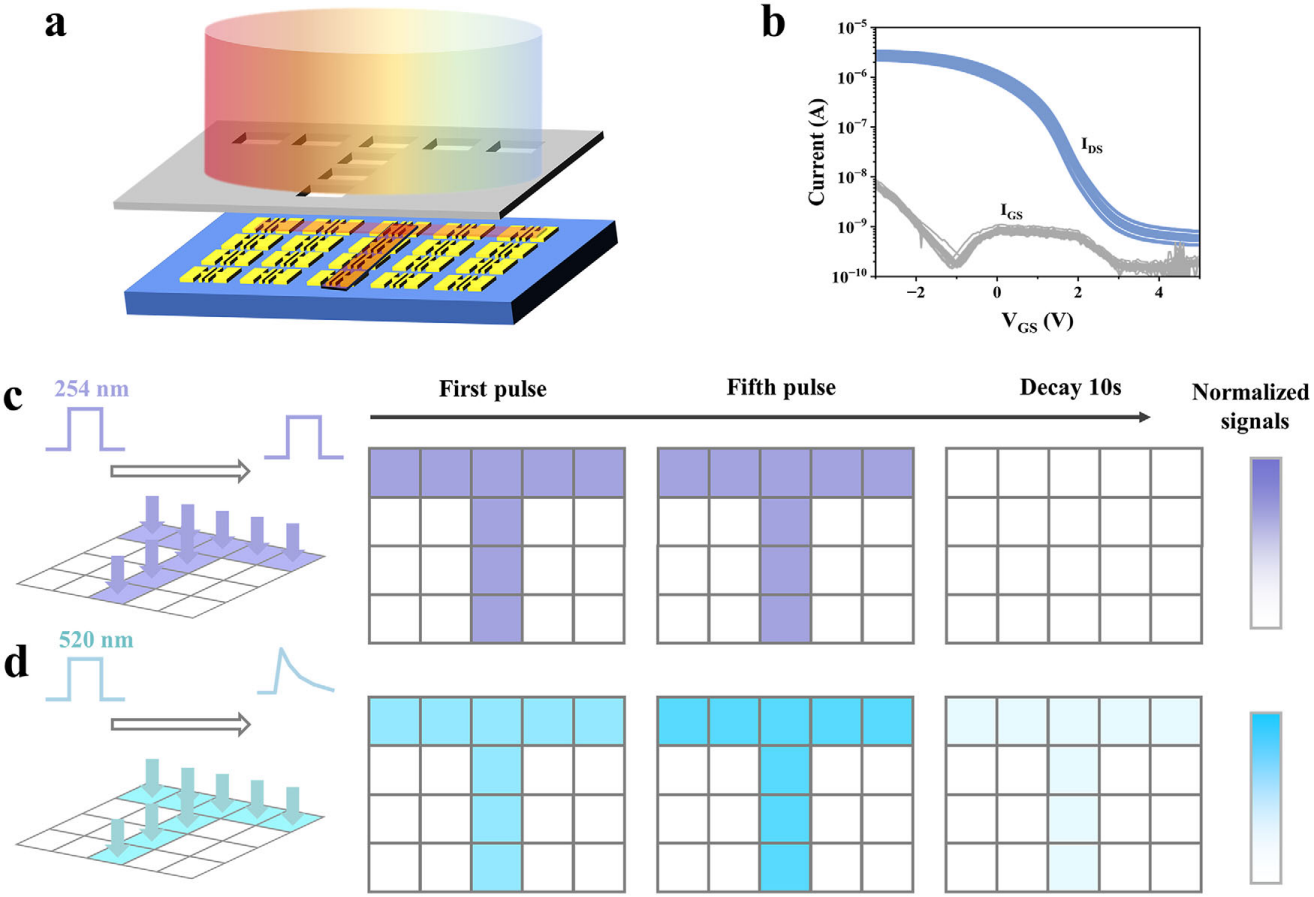
Dual‐mode visual preprocessing in a 4 × 5 pixelated MVBT array. a) Schematic illustration of a “T”‐shaped optical pattern projected onto the 4 × 5 MVBT array through a patterned mask. b) Transfer characteristics of the 4 × 5 pixelated MVBT array measured under dark conditions at a fixed V_DS_ of 3 V. c) Transient reconstruction and rapid decay of the “T” pattern under 254 nm illumination in the two‐terminal mode. d) Pulse‐induced enhancement and temporary retention of the “T” pattern under 520 nm illumination in the three‐terminal mode.

### 2.6 CSNN Fusion Network Based on MVBT for Fingerprint Pattern Recognition

Although convolutional neural networks (CNN) and spiking neural network (SNN) architectures have demonstrated strong capability in various pattern‐recognition tasks, they often encounter challenges related to complex dynamics in deep learning [46]. For example, handling more complex datasets typically requires deeper network architectures or wider feature maps, which substantially increases the number of trainable parameters and training time in CNNs. Moreover, the non‐differentiability of the spike generation process makes it difficult to directly apply standard backpropagation algorithms. Existing methods typically rely on surrogate gradients for approximate training, which can easily cause issues such as vanishing or exploding gradients in deep networks or over extended temporal sequences, thereby affecting recognition accuracy. To address these limitations, we propose an MVBT‐based CSNN hybrid training and simulation architecture.

Figure 7a presents the schematic architecture of the proposed CSNN. In this architecture, the DUV photodetection function of the MVBT serves as the front‐end optical sensing unit, providing sensory inputs for subsequent CNN feature extraction. The simulation framework integrates a CNN coupled with an SNN. To validate the feasibility of the MVBT‐enabled CSNN simulation framework, we conducted a pattern‐recognition task using a fingerprint dataset. First, four feature maps with size 400 × 300 are extracted from the original 400 × 300 pixel image via 3 × 3 convolution kernels. Subsequently, a 5 × 5 pooling operation reduces the spatial dimensions of four feature maps to 80 × 60. After a second round of convolution and pooling, sixteen feature maps with a size of 20 × 20 are ultimately obtained from the original pattern. The resulting 6400 feature signals (16 × 20 × 20) from the sixteen feature maps are then directly fed into a classifier comprising a two‐layer fully connected network. Following the classification layer, the feature outputs are transmitted to the CNN‐SNN conversion layer and converted into inputs for the subsequent spiking neurons. The fingerprint category is finally determined according to the firing timing of the output spiking neurons. Figure 7b displays the weight values of the convolutional kernels employed for fingerprint feature recognition. Figure 7c illustrates the weight distribution of the fully connected layer, reflecting the learned connection strengths in the classification module. Figure 7d depicts the firing timing of the five spiking neurons in the output layer, where different spiking neurons correspond to different finger categories. The final classification is achieved by comparing the firing times, with the earliest firing neuron identifying the recognition category.

**FIGURE 7:**
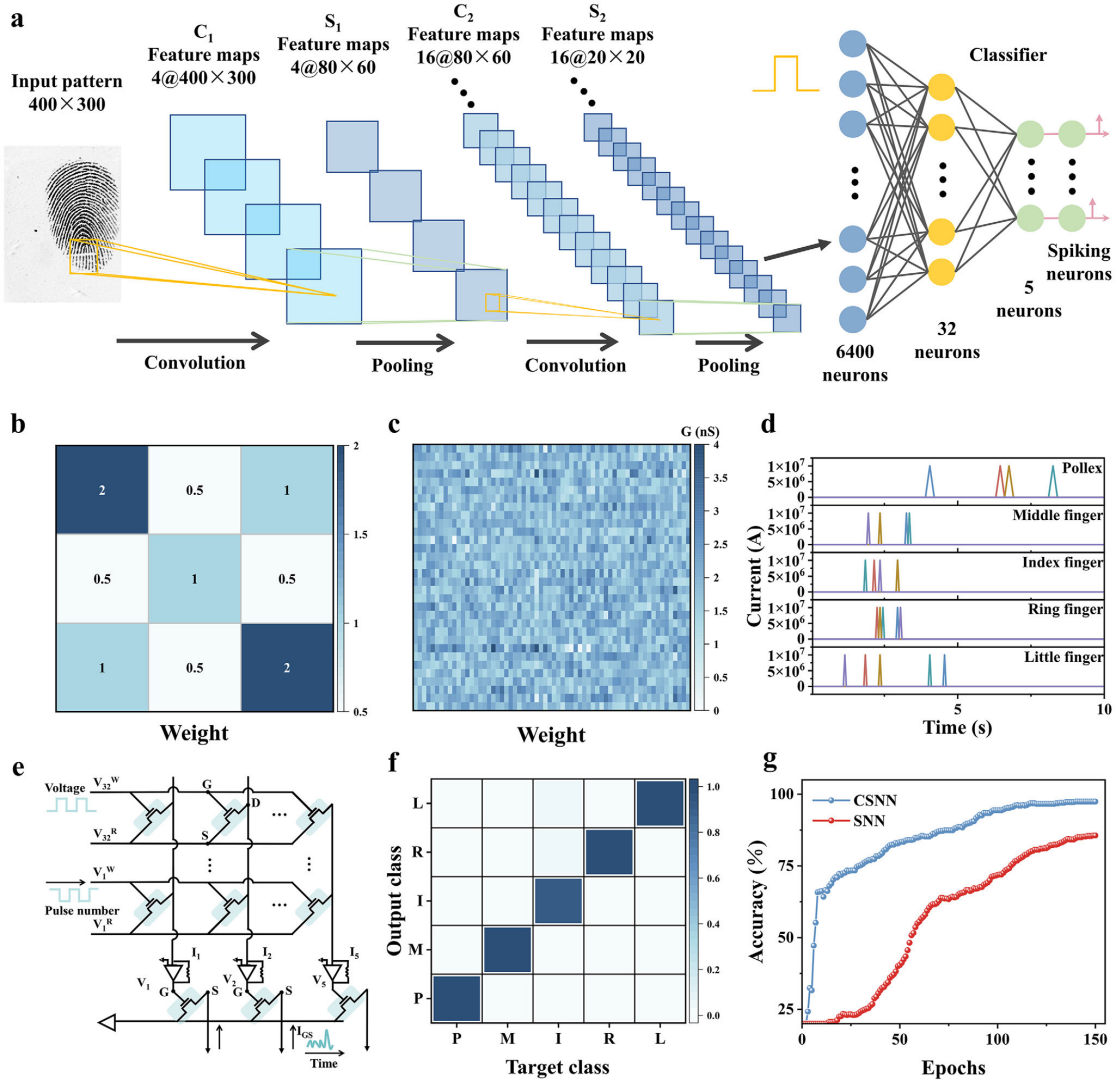
MVBT‐based convolutional spiking neural network for fingerprint recognition. a) Schematic diagram of the CSNN, including the convolutional layer, fully connected layer, and spiking layer. b) The weight values of the convolutional kernels used for fingerprint feature recognition. c) Weight distribution map of the fully connected layer. d) Firing timing diagram of the five spiking neurons in the output layer. e) Circuit schematic of the conversion layer that converts the output of the CNN into the input of the SNN. f) CSNN confusion matrix of the target and output classes for the test results on the fingerprint dataset. g) Recognition accuracy of the SNN and CSNN on the fingerprint dataset.

Figure 7e presents the circuit schematic of the conversion layer between the CNN and SNN. In this layer, the MVBT synaptic units multiply the input feature signals by the corresponding synaptic weights represented by the conductance states and transmit the resulting weighted signals to the neuronal units. The MVBT neuronal units integrate the weighted input signals and generate temporal spikes once the firing threshold is reached, thereby converting the CNN feature outputs into spike‐encoded inputs for the SNN. The confusion matrix for the CSNN on the fingerprint dataset is shown in Figure 7f, comparing the target labels with output labels. Taking the recognition of the index fingerprint as an example, the CSNN successfully identifies it as an index finger with an accuracy of 95%, while misclassifying it as other fingers with a probability of 5%. For comparative analysis, the same fingerprint dataset was trained using an SNN‐based computing architecture, and the corresponding results are summarized in Figure 7g. After 150 training epochs, the proposed CSNN achieves a recognition accuracy of 97.4%, whereas the SNN attains 85.6%. Relative to the SNN, the CSNN exhibits a 13.8% improvement in recognition accuracy. Table S4 summarizes the reported deep neural network architectures based on neuromorphic devices for fingerprint datasets. In comparison with these previously reported devices, the MVBT demonstrates a significant advantage in recognition accuracy. The corresponding loss curves are shown in Figure S20. As the training progresses, the loss value decreases and converges, indicating good training stability and recognition capability for the proposed CSNN architecture.

## 3 Conclusion

In summary, this work presents a multifunctional vertical bulk‐heterojunction transistor with a‐GaO_x_. The device integrates four functions within a single unit: deep‐ultraviolet photodetection, memristive neuron spiking, broadband optoelectronic synaptic behavior, and reconfigurable logic gates. In the two‐terminal (gate–source) operating mode, the ultraviolet‐selective photoresponse and electrochemical reactions of a‐GaO_x_ are exploited to realize deep ultraviolet photodetection and neuronal spike firing, respectively. The device in two‐terminal mode exhibits a peak detectivity of 5.8 × 10^9^ Jones under 254 nm illumination and demonstrates stable threshold‐triggered spiking behavior. In the three‐terminal transistor mode, the organic bulk‐heterojunction active layer enables broadband optoelectronic synaptic functionality, while the asymmetric interfacial carrier‐injection barriers further endow the device with rectifying characteristics and reconfigurable logic‐gate capability. The energy consumption per synaptic event can be as low as 15.7 aJ, and both AND and OR logic functions are successfully realized. In addition, the 4 × 5 pixelated MVBT array enables dual‐mode optical pattern processing, including transient DUV image reconstruction in the two‐terminal mode and pulse‐induced image enhancement and temporary retention in the three‐terminal synaptic mode. Furthermore, a convolutional spiking neural network hybrid simulation architecture based on the MVBT verifies its system‐level potential for complex pattern‐recognition tasks. Therefore, MVBT not only provides a new strategy for the integrated design of multifunctional neuromorphic devices, but also holds important significance for the development of highly function‐dense, low‐redundancy neuromorphic hardware integrating sensing, memory, computing, and decision‐making.

## 4 Experimental Section

### 4.1 Materials Availability

PDVT‐10 and PC_61_BM were procured from Derthon Optoelectronic Material Science and Technology Co., Ltd. PDVT‐10 and PC_61_BM were dissolved at a concentration of 5 mg mL^−1^ in chloroform. Subsequently, the PDVT‐10 solution and PC_61_BM solution were mixed in a volumetric ratio of 10:1 to prepare a solution for the semiconductor layer. Ga(NO_3_)_3_ was purchased from Shanghai Macklin Biochemical Co., Ltd. and dissolved at a concentration of 2 mol L^−1^ in ultrapure water. MXene (5 mg mL^−1^ in deionized water) was obtained from Hangzhou Yanqu Information Technology Co., Ltd. and further diluted to 2 mg mL^−1^ to be used as the source electrode of the MVBT device.

### 4.2 Device Fabrication

The substrates were sequentially cleaned with acetone, isopropanol, and deionized water for 10 min, dried with nitrogen gas, and then subjected to oxygen plasma treatment. Ga(NO_3_)_3_ solution was spin‐coated onto the silicon substrate at a speed of 4500 rpm for 20 s, followed by annealing at 500°C for 1 h, with this process repeated twice to increase the a‐GaO_x_ layer thickness. Subsequently, the diluted MXene solution was spin‐coated onto the a‐GaO_x_ layer at 1000 rpm for 10 s, followed by 2000 rpm for 30 s. Then, 50 nm Ag was thermally evaporated to serve as a contact electrode of the vertical transistor and the top electrode in the neuronal mode. The semiconductor layer was prepared by spin‐coating the PDVT‐10:PC_61_BM solution onto the MXene mesh source electrode at 1000 rpm for 60 s, followed by annealing at 150°C for 15 min. To pattern the semiconductor layer, chloroform etching was employed. Finally, 50 nm of Au was thermally evaporated onto the semiconductor layer as the drain electrode.

### 4.3 Device Characterization

The electrical characteristics and synaptic behavior were investigated using a semiconductor parameter analyzer (Keithley B2912B). The surface morphology of the films was measured using an Atomic Force Microscope (Bruker Dimension Icon). The absorption spectra of the films were measured using a UV–vis spectrophotometer (Shimadzu UV‐2600i). The crystallinity of the a‐GaO_x_ thin films was characterized by X‐ray diffraction (Rigaku SmartLab SE). The surface chemical composition of a‐GaO_x_ was analyzed using X‐ray photoelectron spectroscopy (Thermo Fisher Scientific, USA). Images of the MVBT devices were acquired using a focused ion beam/scanning electron microscope (Sigma 300).

## Author Contributions

**Jinkui Si**: conceptualization, methodology, investigation, writing – original draft. **Qiong Zhuang**: conceptualization, methodology, writing – original draft. **Junling Zhou**: conceptualization. **Lan Yang**: methodology. **Changjie Zhou**: investigation. **Huili Zhu**: supervision. **Minshuai Wang**: formal analysis. **Xiaomei Cai**: formal analysis. **Qiubao Lin**: resources. **Wangying Xu**: writing – review and editing, resources, funding acquisition. **Shuqiong Lan**: writing – review and editing, funding acquisition, resources, investigation. **Rengjian Yu**: software. **Huipeng Chen**: resources, writing – review and editing.

## Funding

The work was supported by National Natural Science Foundation of China (Grant No: 62404083) and Natural Science Foundation of Fujian Province (Grant No: 2023J01331, 2021J05171 and 2026J001808).

## Conflicts of Interest

The authors declare no conflicts of interest.

## Supporting information




**Supporting File**: advs78415‐sup‐0001‐SuppMat.docx.

## Data Availability

The data that support the findings of this study are available from the corresponding author upon reasonable request.
