# Supplementary material for: Multifunctional Vertical Bulk‐Heterojunction Transistors With Amorphous GaOx for Neuromorphic Perception‐to‐Decision
Source: Adv Sci (Weinh). 2026 Oct 20:e78415. Online ahead of print. doi: 10.1002/advs.78415 (PMC13683975; doi:10.1002/advs.78415)
Supplement: Supplementary file 1 [file ADVS-9999-e78415-s001.docx]

Supporting Information

Multifunctional Vertical Bulk-Heterojunction Transistors with Amorphous GaO_x_ for Neuromorphic Perception-to-Decision

Jinkui Si, Qiong Zhuang, Rengjian Yu, Junling Zhou, Lan Yang, Changjie Zhou, Huili Zhu, Minshuai Wang, Xiaomei Cai, Qiubao Lin, Wangying Xu*, Shuqiong Lan*, Huipeng Chen

Jinkui Si, Qiong Zhuang, Junling Zhou, Lan Yang, Changjie Zhou, Huili Zhu, Minshuai Wang, Xiaomei Cai, Qiubao Lin, Wangying Xu, Shuqiong Lan

Department of Physics, School of Science, Jimei University, Xiamen 361021, People’s Republic of China
E-mail: shuqionglan@jmu.edu.cn; wyxu@jmu.edu.cn

Rengjian Yu, Huipeng Chen

Institute of Optoelectronic Display, National & Local United Engineering Lab of Flat Panel Display Technology, Fuzhou University, Fuzhou 350002, People’s Republic of China

Contents

**Figure S1.** Chemical structures of a) PDVT-10 and b) PC_61_BM.

**Figure S2.** MVBT deep-ultraviolet photodetection performance.

**Figure S3.** Control experiments for the 254 nm DUV photoresponse, including the complete MVBT, PC_61_BM-free device, and MXene-free device.

**Figure S4.** Fifty consecutive SET/RESET switching cycles and corresponding V_SET_ and V_RESET_ statistics.

**Figure S5.** Ag-nanowire-mesh source-electrode control device and its threshold-switching characteristics.

**Figure S6.** Current response of the MVBT neuron channel stimulated by −2.5 V pulse.

**Figure S7.** Leakage process in MVBT neuron channel.

**Figure S8.** Finite-element simulation of potential distribution between gate and source.

**Figure S9.** Transfer characteristics of the MVBT under negative bias.

**Figure S10.** PPF and PPD responses of the MVBT.

**Figure S11.** PPF and PPD test curves at various intervals.

**Figure S12.** Fitted PPF curves under illumination at different wavelengths.

**Figure S13.** The EPSC response of the device under 365 nm illumination with an intensity of 0.18 mW cm^−2^.

**Figure S14.** Learning process simulated using light pulses of different wavelengths.

**Figure S15.** Current–time responses of the MVBT under 5, 10, 20, 40, and 60 consecutive electrical pulses: a) excitatory and b) inhibitory responses.

**Figure S16.** Training conductance under illumination at different wavelengths.

**Figure S17.** Energy band diagrams of the MVBT in potentiation, depression, and illumination modes.

**Figure S18.** Simulated current density distribution under positive and negative gate bias.

**Figure S19.** I_DS_ mapping as a function of V_DS_ and V_GS_.

**Figure S20.** Loss functions of SNN and CSNN on the Fingerprint Dataset.

**Figure S21.** Device-to-device variation of the 4 × 5 MVBT array.

**Table S1.** Performance comparison of GaO_x_-based ultraviolet photodetectors in recent years.

**Table S2.** Comparison of MXene with other alternatives source electrode.

**Table S3.** Comparison of the reported energy consumption in photoelectric synapses.

**Table S4.** Comparison of representative neuromorphic devices for fingerprint recognition.

**Text S1.** Migration and diffusion of Ag^+^ in MVBT neuronal channels.

**Figure S1.** Chemical structures of a) PDVT-10 and b) PC_61_BM.

**Figure S2.** MVBT deep-ultraviolet photodetection performance. a) Response time of the device under 254 nm light irradiation at V_GS_ = 4 V. b) Photocurrent (I_ph_ = I_light_ − I_dark_) as a function of incident light power density at V_GS_ = 4 V. c) Responsivity and specific detectivity as a function of optical power density at V_GS_ = 4 V.

**Figure S3.** Control experiments for the 254 nm DUV photoresponse. I_GS_–V_GS_ characteristics measured under dark conditions and 254 nm illumination for a) the complete MVBT, b) the PC_61_BM-free device, and c) the MXene-free device. All devices were measured under identical electrical conditions and 254 nm illumination with a power density of 1.32 mW cm^−2^.

**Figure S4.** Cycle-to-cycle threshold-switching stability of a representative MVBT. a) Fifty consecutive SET/RESET switching cycles measured in the gate–source mode. b,c) Statistical distributions of the corresponding b) V_SET_ and c) V_RESET_ values over 50 switching cycles. The mean values (μ) and standard deviations (σ) are indicated.

**Figure S5.** Ag-nanowire-mesh source-electrode control device and its threshold-switching characteristics. a) Structural diagram of the MVBT neuronal channel using an Ag-nanowire mesh source electrode. b) I_GS_-V_GS_ characteristics of the Ag-nanowire-mesh control device.

**Figure S6.** Current response of the MVBT neuron channel stimulated by −2.5 V pulse.

**Figure S7.** Leakage process in MVBT neuron channel.

**Figure S8.** Finite-element simulation of potential distribution between gate and source electrode. a) V_GS_ = 1 V. b) V_GS_ = 2 V. c) V_GS_ = 3 V. d) V_GS_ = −1 V. e) V_GS_ = −2 V. f) V_GS_ = −3 V.

**Figure S9.** Transfer characteristics of the MVBT under negative bias.

**Figure S10.** a) PPF and b) PPD responses of the MVBT (V_GS_ = ±2 V, 20 ms).

**Figure S11.** PPF and PPD test curves at various intervals.

**Figure S12.** Fitted PPF curves under illumination at different wavelengths. a) 365 nm. b) 520 nm. c) 808 nm.

**Figure S13.** The EPSC response of the device under 365 nm illumination with an intensity of 0.18 mW cm^−2^ (V_DS_ = 1 μV).

**Figure S14.** Learning process simulated using light pulses of different wavelengths (0.18 mW cm^−2^, V_DS_ = −2 V, 0.2 s). a) 365 nm. b) 520 nm. c) 808 nm.

**Figure S15.** Current-time responses of the MVBT under 5, 10, 20, 40, and 60 consecutive electrical pulses: a) excitatory and b) inhibitory responses.

**Figure S16.** Training conductance under illumination at different wavelengths (0.18 mW cm^−2^, V_DS_ = −2 V, 0.2 s). a) Dark. b) 365 nm. c) 520 nm. d) 808 nm.

**Figure S17.** Energy band diagrams of the MVBT in a) potentiation, b) depression, and c) illumination modes.

**Figure S18.** Simulated current density distribution under positive and negative gate bias. a) The 2D cross-section simulation model of simplified MVBT performed in COMSOL Multiphysics. b) and c) show the current density profiles along the red line in a) after applying gate voltages of 3 V and −3 V, respectively.

**Figure S19.** I_DS_ mapping as a function of V_DS_ and V_GS_. a) Under dark conditions. b) Under 365 nm illumination. c) Under 520 nm illumination. d) Under 940 nm illumination (0.18 mW cm^−2^).

**Figure S20.** Loss functions of SNN and CSNN on the Fingerprint Dataset.

**Figure S21.** Device-to-device variation of the 4 × 5 MVBT array. a) Statistical distribution of the threshold voltage V_th_ extracted from 20 MVBT devices, with an average value of 2.025 V and a standard deviation of 0.125 V. b) Spatial distribution of the ON/OFF current ratio across the 20 MVBT devices in the 4 × 5 array.

**Table S1.** Performance comparison of GaO_x_-based ultraviolet photodetectors in recent years.

| **Device structure** | **λ** | **Dark current** | **Responsivity** | **Detectivity** | | **Rise/decay time** | **Ref.** |
| --- | --- | --- | --- | --- | --- | --- | --- |
| Pt/a-Ga_2_O_3_/ITO | 254 nm | 10 pA (−3 V) | 3.69 A/W | | 3.1 × 10^12^ Jones | 2.6/6.6 ms | ^1^ |
| Au/a-Ga_2_O_3_/ITO | 250/275 nm | 200 pA (0 V) | 0.014 A/W | | ~4 × 10^11^ Jones | ~2/~2 μs | ^2^ |
| Au/β-Ga_2_O_3_ nanowire | 270 nm | 10 pA (−30 V) | 10 × 10^−5^ A/W | | - | 1/64 μs | ^3^ |
| Ag NWs/a-Ga_2_O_3_ | 254 nm | 10^5^ pA (0 V) | 2.3 × 10^−7^ A/W | | 3.1 × 10^9^ Jones | 240 ms | ^4^ |
| Pt/β-Ga_2_O_3_/C | 310/325 nm | 100 pA (−10 V) | 0.011 A/W | | 3.7 × 10^10^ Jones | 15/30 ms | ^5^ |
| MLG/β-Ga_2_O_3_/FTO | 230/250 nm | 50 pA (0 V) | 0.0092 A/W | | 5.27 × 10^11^ Jones | 2/8.8 ms | ^6^ |
| Ga_2_O_3_ microwire/MXene | 254 nm | 0.1-1 pA (0 V) | 2.5 × 10^−4^ A/W | | 4 × 10^9^ Jones | 0.162/35.8 ms | ^7^ |
| Ti_3_C_2_T_x_ MXene/α-Ga_2_O_3_ | 254 nm | 0.41 pA (−5 V) | 0.0311 A/W | | - | 48.5/41.7 μs | ^8^ |
| PEDOT:PSS/α-Ga_2_O_3_/rh-ITO | 220 nm | - | 9.7 × 10^−4^ A/W | | - | - | ^9^ |
| **Ag/MXene/a-GaO_x_/p-Si** | **254 nm** | **69 pA (0 V)** | **0.278 A/W (4 V)**  **0.2 A/W (0 V)** | | **5.8 × 10^9^ Jones** | **36.14/17.55 ms** | **This work** |

**Table S2.** Comparison of MXene with other alternatives source electrode.

| **Gate dielectric materials** | **Source electrode** | **Semiconductor** | **I_on_/I_off_** | | **V_th_** | **Scanning range** | **Ref.** |
| --- | --- | --- | --- | --- | --- | --- | --- |
| SiO_2_ | CNT | MoS_2_ | | 10^3^ | >-40 V^a^ | 60 to −60 V | ^10^ |
| SiO_2_ | Grahphene | C_60_ | | 3 × 10^3^ | >50 V^a^ | 150 to −50 V | ^11^ |
| SiO_2_ | Grahphene | Pentacene | | <10^3^ | >30 V^a^ | 80 to −80 V | ^12^ |
| Al_2_O_3_ | Pattern Au | CuPc | | 10 | 1 | 0 to −1.5 V | ^13^ |
| Al_2_O_3_ | AgNWs | PDVT-8 | | 2 × 10^4^ | 1.5 | 40 to −40 V | ^14^ |
| TaO_x_ | MXene | PDVT-10:N2200 | | 1 × 10^3^ | 1.1 | 8 to −6 V | ^15^ |
| **a-GaO_x_** | **MXene** | **PDVT-10:PC_61_BM** | | **5 × 10^3^** | **2** | **6 to −4 V** | **This work** |

^a^ estimated from the transfer curves of reported devices.

**Table S3.** Comparison of the reported energy consumption in photoelectric synapses.

| **Device structure** | **Minimum light intensity**  **(mW cm^−2^)** | **Operation type** | | **Energy**  **consumption (fJ)** | **Ref.** |
| --- | --- | --- | --- | --- | --- |
|  |  | Excitatory | Inhibitory |  |  |
| Si NCs | 30 | V_GS_ | 1342 nm | 9.14 × 10^6^ | ^16^ |
| Pentacene/PMMA/C_3_N_4_ | 0.06 | 365 nm | - | 18 | ^17^ |
| ITZO | - | 400 nm | V_GS_ | 2.5 × 10^6^ | ^18^ |
| IGZO/ZrO_x_ | 0.87 | 405 nm | 890 nm | 2.3 × 10^10^ | ^19^ |
| Graphene/TiO_2_ QD | 26 | 635 nm | 365 nm | 1.0 × 10^7^ | ^20^ |
| P3HT/CsPbBr_3_ QD | 10 | 445 nm | V_GS_ | 2.5 × 10^8^ | ^21^ |
| Pentacene/PMMA/  Zr-CsPbI_3_ | 0.012 | 405 nm | V_GS_ | 1.0 × 10^8^ | ^22^ |
| IDTBT/ITO | 0.096 | 450 nm | 450 nm | 2.4 | ^23^ |
| Graphene/Rubrene | - | 365 nm | - | 5.8 × 10^−2^ | ^24^ |
| PVP/ZrO_x_ | - | V_GS_ | - | 1.43 | ^25^ |
| PEA_2_SnI_4_/PEIE/C_60_ | - | V_GS_ | - | 7.3 × 10^6^ | ^26^ |
| Bi_2_Se_3_ | 30.2 | 532 nm | - | 9.2 | ^27^ |
| Perylene/Honey | 0.00043 | 482 nm | - | 2.4 | ^28^ |
| InSe | 87.83 | 650 nm | - | 8 | ^29^ |
| **PDVT-10/PC_61_BM** | **0.18** | **365 nm** | **V_GS_** | **1.57 × 10^−2^** | **This work** |

**Table S4.** Comparison of representative neuromorphic devices for fingerprint recognition.

| **Structure** | **Additional functional layer** | **Material system** | **Dataset** | | **Recognition accuracy** | **Ref.** |
| --- | --- | --- | --- | --- | --- | --- |
| Vertical | PyMPM trapping layer | 2D perovskite / organic semiconductor | | Fingerprint dataset | 96.7% | ^30^ |
| Planar | Al_2_O_3_ encapsulation | Ga_2_O_3_ / Al_2_O_3_ | | Fingerprint dataset | 91.8% | ^31^ |
| Planar | None | Mixed-halide perovskite / Y_2_O_3_:Eu^3+^ | | Fingerprint dataset | 94.4% | ^32^ |
| Vertical | None | Polymer memristor | | Fingerprint dataset | 97.15% | ^33^ |
| Planar | Bilayer dielectric | DNTT / pBDDA / Parylene | | Fingerprint dataset | 96% | ^34^ |
| Planar | None | OECT array | | Fingerprint dataset | 95.0% | ^35^ |
| **Vertical** | **None** | **PDVT-10:PC_61_BM** | | **Fingerprint dataset** | **97.4%** | **This work** |

**Text S1.** Migration and diffusion of Ag^+^ in MVBT neuronal channels.

The migration process of Ag^+^ in the neuronal channel can be characterized by two coupled regions: the MXene region and the GaO_x_ region. Previous studies have demonstrated that conductive filaments tends to preferentially grow along MXene nanostructures, thereby reducing the randomness of filament formation and improving device performance.^[36]^ Moreover, Ag^+^ migration on MXene-related surfaces has been reported in Ag/MXene/SiO_2_/Si artificial neural devices.^[37]^ In the oxide region, recent studies on Ag/Ga_2_O_3_/Pt memristors indicate that Ag^+^ transport in gallium oxide contributes to resistive switching, especially in amorphous Ga_2_O_3_ films.^[38]^ Therefore, the diffusion behavior of Ag^+^ in the present MXene/GaO_x_ neuronal channel can be described phenomenologically by an over-damped Langevin-type equation:^[39]^

$$\begin{aligned} \eta dr/dt=-\nabla U(r)+q_{eff}E(t)+F_{th}(t)\#(S1) \end{aligned}$$

where η is the effective damping coefficient, 𝐫 denotes the position vector of Ag^+^, U(𝐫) is the local potential-energy landscape, q_eff_ E(t) represents the electric-field-driven force, and F_th_(t) accounts for thermally activated stochastic fluctuation. Within this framework, the potential term reflects the energetic inhomogeneity arising from interfaces, MXene edges and defects in GaO_x_. The electric-field term describes the bias-driven drift of Ag^+^, and the thermal term encompasses both random hopping under bias and relaxation following bias removal. Upon application of a bias, Ag is oxidized into Ag^+^ and migrates through the GaO_x_ region under the combined influence of drift and diffusion. Local accumulation of Ag^+^ at energetically favorable sites can trigger the formation of conductive pathways, leading to an increase in conductance. After the bias is removed, the ionic distribution evolves toward a lower-energy configuration, which can lead to thinning, rupture or partial dissolution of the conductive pathway.

References

1. L. Ye, S. Zhou, Y. Xiong, et al., “Self-powered Pt/a-Ga_2_O_3_/ITO vertical schottky junction solar-blind photodetector with excellent detection performance,” *Optics Express* 31, no. 17 (2023): 28200-28211. https://doi.org/10.1364/OE.494216

2. A. K. Das, V. K. Sahu, R. S. Ajimsha, et al., “Ultrafast self-powered solar blind UV photodetectors based on amorphous Ga_2_O_3_ thin films in crossbar geometry,” *Materials Today Physics* 60 (2026): 102007. https://doi.org/10.1016/j.mtphys.2025.102007

3. X. Chen, K. W. Liu, Z. Z. Zhang, et al., “Self-powered solar-blind photodetector with fast response based on Au/β-Ga_2_O_3_ nanowires array film schottky junction,” *ACS Applied Materials & Interfaces* 8, no. 6 (2016): 4185-4191. https://doi.org/10.1021/acsami.5b11956

4. S. Wang, C. Wu, F. Wu, et al., “Flexible transparent and self-powered deep ultraviolet photodetector based on Ag NWs/amorphous gallium oxide Schottky junction for wearable devices,” *Sensors and Actuators A: Physical* 330 (2021): 112870. https://doi.org/10.1016/j.sna.2021.112870

5. S. Sharma, P. Y. Le, M. W. Allen, et al., “RF sputtered Ga_2_O_3_ thin-films for solar-blind UV-C detection: Investigating effects of crystallinity, stoichiometry, and ambient,” *APL Electronic Devices* 1, no. 3 (2025): 036129. https://doi.org/10.1063/5.0293906

6. Y. Li, Z. Zhou, H. Pan, et al., “High-performance Ga_2_O_3_/FTO-based self-driven solar-blind UV photodetector with thickness-optimized graphene top electrode,” *Journal of Materials Research and Technology* 22 (2023): 2174-2185. https://doi.org/10.1016/j.jmrt.2022.12.086

7. Y. Liu, Y. Wei, S. Sha, et al., “Flexible self-powered solar-blind Schottky photodetectors based on individual Ga_2_O_3_ microwire/MXene junctions,” *CrystEngComm* 25, no. 37 (2023): 5324-5333. https://doi.org/10.1039/D3CE00620D

8. J. Cao, Y. Guan, H. Chen, et al., “High-performance solar-blind UV photodetector based on Ti_3_C_2_T_x_ MXene/α-Ga_2_O_3_ heterojunction,” *Applied Physics Letters* 128, no. 13 (2026): 133301. https://doi.org/10.1063/5.0323532

9. K. Shimazoe, H. Nishinaka, Y. Taniguchi, et al., “Vertical self-powered ultraviolet photodetector using α-Ga_2_O_3_ thin films on corundum structured rh-ITO electrodes,” *Materials Letters* 341 (2023): 134282. https://doi.org/10.1016/j.matlet.2023.134282

10. T. L. Phan, Q. A. Vu, Y. R. Kim, et al., “Efficient gate modulation in a screening-engineered MoS_2_/single-walled carbon nanotube network heterojunction vertical field-effect transistor,” *ACS Applied Materials & Interfaces* 11, no. 28 (2019): 25516-25523. https://doi.org/10.1021/acsami.9b05335

11. K. Kim, T. H. Lee, E. J. G. Santos, et al., “Structural and electrical investigation of C_60_-graphene vertical heterostructures,” *ACS Nano* 9, no. 6 (2015): 5922-5928. https://doi.org/10.1021/acsnano.5b00581

12. J. S. Kim, B. J. Kim, Y. J. Choi, et al., “An organic vertical field-effect transistor with underside-doped graphene electrodes,” *Advanced Materials* 28, no. 24 (2016): 4803-4810. https://doi.org/10.1002/adma.201505378

13. A. Nawaz, L. Merces, D. M. de Andrade, et al., “Edge-driven nanomembrane-based vertical organic transistors showing a multi-sensing capability,” *Nature Communications* 11, no. 1 (2020): 841. https://doi.org/10.1038/s41467-020-14661-x

14. Y. Fang, X. Wu, S. Lan, et al., “Inkjet-printed vertical organic field-effect transistor arrays and their image sensors,” *ACS Applied Materials & Interfaces* 10, no. 36 (2018): 30587-30595. https://doi.org/10.1021/acsami.8b06625

15. Y. Zou, D. Liu, X. Gan, et al., “Toward switching and fusing neuromorphic computing: vertical bulk heterojunction transistors with multi‐neuromorphic functions for efficient deep learning,” *Advanced Materials* 37, no. 27 (2025): 2419245. https://doi.org/10.1002/adma.202419245

16. L. Yin, C. Han, Q. Zhang, et al., “Synaptic silicon-nanocrystal phototransistors for neuromorphic computing,” *Nano Energy* 63 (2019): 103859. https://doi.org/10.1016/j.nanoen.2019.103859

17. H. L. Park, H. Kim, D. Lim, et al., “Retina-inspired carbon nitride-based photonic synapses for selective detection of UV light,” *Advanced Materials* 32, no. 11 (2020): e1906899. https://doi.org/10.1002/adma.201906899

18. J. Kim, S. Song, H. Kim, et al., “Light-stimulated artificial photonic synapses based on solution-processed In-Sn-Zn-O transistors for neuromorphic applications,” *Journal of Alloys and Compounds* 903 (2022): 163873. https://doi.org/10.1016/j.jallcom.2022.163873

19. Y. C. Mi, C. H. Yang, L. C. Shih, et al., “All-optical-controlled excitatory and inhibitory synaptic signaling through bipolar photoresponse of an oxide-based phototransistor,” *Advanced Optical Materials* 11, no. 14 (2023): 2300089. https://doi.org/10.1002/adom.202300089

20. J. Liang, X. Yu, J. Qiu, et al., “All-optically controlled artificial synapses based on light-induced adsorption and desorption for neuromorphic vision,” *ACS Applied Materials & Interfaces* 15, no. 7 (2023): 9584-9592. https://doi.org/10.1021/acsami.2c20166

21. G. K. Gupta, I. J. Kim, Y. Park, et al., “Inorganic perovskite quantum dot-mediated photonic multimodal synapse,” *ACS Applied Materials & Interfaces* 15, no. 14 (2023): 18055-18064. https://doi.org/10.1021/acsami.2c23218

22. H. Shao, Y. Li, W. Yang, et al., “A reconfigurable optoelectronic synaptic transistor with stable Zr-CsPbI_3_ nanocrystals for visuomorphic computing,” *Advanced Materials* 35, no. 12 (2023): 2208497. https://doi.org/10.1002/adma.202208497

23. C. Liu, C. Gao, W. Huang, et al., “Bipolar synaptic organic/inorganic heterojunction transistor with complementary light modulation and low power consumption for energy-efficient artificial vision systems,” *Science China Materials* 67, no. 5 (2024): 1500-1508. https://doi.org/10.1007/s40843-024-2812-7

24. K. Chen, Q. Dai, Y. Tian, et al., “Ultra‐low‐power vertical organic synaptic phototransistors for neuromorphic vision preprocessing,” *Advanced Functional Materials* 35, no. 48 (2025): 2508673. https://doi.org/10.1002/adfm.202508673

25. Y. Li, W. Cai, R. Tao, et al., “Flexible and energy-efficient synaptic transistor with quasi-linear weight update protocol by inkjet printing of orientated polar-electret/high-k oxide composite dielectric,” *ACS Applied Materials & Interfaces* 16, no. 15 (2024): 19271-19282. https://doi.org/10.1021/acsami.4c02880

26. Y. T. Yang, Y. H. Shih, Q. G. Chen, et al., “Revealing the potential of perovskite transistors for dual-modulated synaptic behavior through heterojunction design,” *ACS Energy Letters* 9, no. 9 (2024): 4564-4571. https://doi.org/10.1021/acsenergylett.4c01784

27. S. Nandi, S. Ghosal, M. Meyyappan, et al., “Defect-engineered 2D Bi_2_Se_3_-based broadband optoelectronic synapses with ultralow energy consumption for neuromorphic computing,” *Materials Horizons* 12, no. 12 (2025): 4274-4288. https://doi.org/10.1039/D4MH01625D

28. T. Serghiou, J. D. Fernandes, V. Karthikeyan, et al., “Sustainable and tunable synaptic electrolyte-gated organic field-effect transistors (EGOFETs) for light adaptive visual perceptive systems,” *Advanced Functional Materials* 35, no. 11 (2025): 2417355. https://doi.org/10.1002/adfm.202417355

29. M. Li, H. Chu, C. Gao, et al., “Bioinspired high-order in-sensor spatiotemporal enhancement in van der Waals optoelectronic neuromorphic electronics,” *Nature Communications* 16, no. 1 (2025): 8801. https://doi.org/10.1038/s41467-025-63873-6

30. C. Xie, C. Li, H. Yu, et al., “Electron trapping enhanced deep-ultraviolet optoelectronic synapses based on two-dimensional wide-bandgap perovskite/organic-semiconductor heterojunctions,” *Nano Letters* 25, no. 40 (2025): 14686-14694. https://doi.org/10.1021/acs.nanolett.5c03851

31. P. Li, J. Shi, Z. Xu, et al., “Boosting performance of Ga_2_O_3_ thin-film transistors via defect passivation toward solar-blind ultraviolet in-sensor reservoir computing,” *ACS Nano* 20, no. 1 (2026): 1420-1430. https://doi.org/10.1021/acsnano.5c18136

32. Y. Zhao, M. Li, M. C. Wong, et al., “Smart phosphor with neuromorphic behaviors enabling full-photoluminescent Write and Read for all-optical physical reservoir computing,” *Nature Communications* 16, no. 1 (2025): 7516. https://doi.org/10.1038/s41467-025-62745-3

33. J. Zhou, W. Li, Y. Chen, et al., “Optoelectronic polymer memristors with dynamic control for power-efficient in-sensor edge computing,” *Light: Science & Applications* 14, no. 1 (2025): 309. https://doi.org/10.1038/s41377-025-01986-9

34. G. Lee, S. Jeong, H. Kim, et al., “Photoresponsive dual-mode memory transistor for optoelectronic computing: charge storage and synaptic signal processing,” *npj Flexible Electronics* 9, no. 1 (2025): 65. https://doi.org/10.1038/s41528-025-00444-1

35. D. Yang, J. Wang, M. Xu, et al., “An inkjet-printable organic electrochemical transistor array with differentiated ion dynamics for sweat fingerprint identification,” *Device* 3, no. 4 (2025): 100651. https://doi.org/10.1016/j.device.2024.100651

36. X. Lian, X. Shen, M. Zhang, et al., “Resistance switching characteristics and mechanisms of MXene/SiO_2_ structure-based memristor,” *Applied Physics Letters* 115, no. 6 (2019): 063501. https://doi.org/10.1063/1.5087423

37. B. Zeng, X. Zhang, C. Gao, et al., “MXene-based memristor for artificial optoelectronic neuron,” *IEEE Transactions on Electron Devices* 70, no. 3 (2023): 1359-1365. https://doi.org/10.1109/TED.2023.3234881

38. H. J. Lee, J. H. Kim, H. J. Kim, et al., “Improvements in resistive and capacitive switching behaviors in Ga_2_O_3_ memristors via high-temperature annealing process,” *Materials* 17, no. 11 (2024): 2727. https://doi.org/10.3390/ma17112727

39. Z. Wang, S. Joshi, S. E. Savel'ev, et al., “Memristors with diffusive dynamics as synaptic emulators for neuromorphic computing,” *Nature Materials* 16, no. 1 (2017): 101-108. https://doi.org/10.1038/nmat4756
